# Effect of different sources of selenium supplementation on immune function in pregnant and lactating ewes

**DOI:** 10.1186/s40104-025-01311-9

**Published:** 2025-12-25

**Authors:** Rebecka Sadler, Nicole Moran, Umesh K. Shandilya, Eduardo Ribeiro, Bonnie A. Mallard, Amir Bazrgar, Niel A. Karrow

**Affiliations:** 1 Department of Animal Biosciences, University of Guelph, Guelph, ON N1G 2W1 Canada; 2 ImmunoCeutica Inc, Cambridge, ON N1T 1N6 Canada; 3 Department of Pathobiology, University of Guelph, Guelph, ON N1G 2W1 Canada; 4 Ontario Agricultural College, University of Guelph, Guelph, ON N1G 2W1 Canada

**Keywords:** Immune function, Ovine lactation, Ovine pregnancy, Selenium supplementation

## Abstract

**Background:**

Selenium (Se) is an essential soil mineral that can be incorporated into animal feedstuffs. Because of a lack of soil Se in some regions, organic or inorganic supplementation strategies must be implemented to prevent deficiencies and promote optimal ovine health. Therefore, the objectives of this study were to assess how inorganic versus organic Se supplementation influenced ewe Se status and immune function during late gestation and postpartum. Dorset Rideau ewes (*n* = 110) were fed a Se deficient diet from gestation d 110 through postpartum d 49 and received one of four daily oral Se treatments diluted in 5 mL of sugar water: 0 mg Se, 0.3 mg inorganic Se, 0.3 mg organic Se, and 0.6 mg organic Se. Throughout the trial, the ewes received various immune challenges, including intramuscular immunizations with a novel antigen (ovalbumin; OVA) on trial d 0 and 10, an intradermal OVA challenge on d 20, and a lipopolysaccharide (LPS) endotoxin challenge on trial d 49.

**Results:**

The organic Se treatment groups had higher serum Se concentrations on most trial days compared to the 0.3 mg inorganic and control groups (*P* < 0.05). No significant treatment differences were found for the dermal hypersensitivity response to OVA, anti-OVA antibody response, glutathione peroxidase activity, cytokine response, cortisol response, or rectal temperature (*P* > 0.05). However, 4 h post-LPS injection, the serum albumin concentration was significantly lower in the 0.3 mg inorganic group compared to both organic Se groups, potentially indicating a higher degree of inflammation in the ewes supplemented with the inorganic Se.

**Conclusions:**

The results of this study indicate that organic Se supplementation can promote a higher Se status in ewes over time, but Se supplementation during this study period did not affect tested immunological parameters. This lack of difference in immune responsiveness between groups may be due to an absence of true serum Se deficiencies in the Se-deficient group or the levels of Se supplementation being insufficient to significantly improve immunocompetence.

**Supplementary Information:**

The online version contains supplementary material available at 10.1186/s40104-025-01311-9.

## Introduction

Selenium (Se) is an essential micronutrient for livestock and is critical to support their health and production. Since Se is in the same periodic table group as sulfur (S), thus sharing some of its chemical properties, Se can replace S in certain amino acids, namely methionine and cysteine, to produce selenomethionine (SeMet) and selenocysteine (SeCys) [1]. SeCys is incorporated into bioactive selenoproteins with critical enzymatic functions, such as thyroid hormone metabolism, reduction of oxidative stress, degradation of misfolded proteins, and cellular signaling, while SeMet can be non-selectively incorporated into proteins throughout the body as a Se reservoir, presumably accessed during times of nutritional insufficiency [2]. While there are various biologically relevant selenoproteins, one well-known selenoprotein that functions in an antioxidant capacity is glutathione peroxidase (GPx) [3, 4].

One key motivation for studying the impact of Se supplementation is due to the vast global agricultural regions that are deficient in Se [5]. Moreover, climate change may negatively impact soil Se levels in the future by altering Se’s biogeochemical cycles, potentially challenging the supply of dietary Se to animals and people [6]. If soil is lacking Se, crops subsequently grown in that soil and used for feedstuffs will also be Se deficient [6]. In Ontario, Canada, where the current study took place, many agricultural regions produce feedstuffs deficient in Se (< 0.05 ppm dry matter, DM), highlighting the necessity of finding viable supplement options to prevent Se deficiency [7]. Current industry practice, as directed by the Ontario Ministry of Agriculture, Food and Agribusiness, is to inject newborn lambs either subcutaneously or intramuscularly with an inorganic Se and vitamin E preparation due to health issues of lambs that have been attributed to Se deficiency [8]. However, consistently supplementing dams with enough dietary Se to maintain an optimal Se status and adequate immunity may be a better solution to promote the health of both dam and fetus. Specifically, dam Se deficiency can adversely impact fetal development and selenoprotein expression in neonates, which can enhance risks for morbidities and mortalities [9]. Whereas, Se supplementation to deficient ewes can benefit maternal health parameters, as well as organ and placental development, supporting fetal and neonate health in a more comprehensive way that a postpartum injection cannot [9–11].

A debate continues in the animal agriculture industry regarding the health benefits of providing animals different sources of trace minerals, namely organic versus inorganic types [12–14]. While inorganic Se is reportedly less bioavailable due to the ruminal microbial degradation of Se salts into nonabsorbable selenospecies, organic Se may have higher absorption rates; however, a cost-benefit analysis for each operation should be performed due to the higher cost of organic Se supplementation [15]. Thus, providing further clarity to this area of research was another important reason for studying organic versus inorganic of Se as a part of the current study.

Finally, previous research has highlighted the essentiality of Se to ovine health and immunity [16–19]. Immunological parameters that Se has reportedly improved in sheep include, antibody titers, antioxidant status, production of interferon (IFN)-γ, gamma globulin levels, lysozyme activity, respiratory burst activity, and T-cell proliferation [19–21]. In other agricultural animals, Se also has immune system benefits as indicated by enhanced immunoglobulin (Ig) production, macrophage phagocytic activity, natural killer (NK) cell cytotoxicity, disease resistance, passive immunity transfer to offspring, and antioxidant status [22, 23]. However, not all studies evaluating the effects of Se supplementation in livestock have shown significant improvements in immune responses [24], while other studies have shown that a high level of Se supplementation can cause immunosuppression and other toxic side effects [25]. These existing contradictions in the literature highlight the need for studies assessing the immunological effects of Se to determine what levels of Se supplementation are required to promote optimal ewe health during pregnancy and lactation.

Therefore, the objectives of this study were to determine how organic and inorganic Se supplementation affected ewe Se status, as well as innate and adaptive immune responses. The hypotheses were that the organic Se treatment would yield a higher Se status in comparison to the inorganic Se treatment with the 0.6 mg organic Se treatment group having a higher Se status than the 0.3 mg organic Se treatment group. It was also hypothesized that the organic Se treatment groups would have increased adaptive immune and an attenuated inflammatory acute-phase responses in comparison to the inorganic group with the 0.6 mg organic group having a heightened adaptive immune and lowered acute phase response in comparison to the 0.3 mg organic group.

## Materials and methods

The University of Guelph Animal Care Committee approved this study’s experimental protocol under Animal Utilization Protocol #4693. The study was conducted at the Ontario Sheep Research Centre (Ponsonby, ON, Canada).

### Se sources

The organic Se was in the form of 2% hydroxy-selenomethionine and was provided by Adisseo (Antony, France). The inorganic Se from Aurubis (Hamburg, Germany) was in the form of 1% sodium selenite (Na_2_SeO_3_) with 36% calcium carbonate and was also provided by Adisseo.

### Animals and experimental design

On gestation d 110 (GD 110 or trial d 0), 110 healthy Dorset-Rideau ewes aged 2–5 years were enrolled in a randomized complete block design at the Ontario Sheep Research Centre. Ewes were enrolled in a period of two years to one of five blocks of approximately 25 sheep with similar pregnancy dates. In brief, the ewes were estrus synchronized with a protocol starting with the implantation of intravaginal controlled internal drug release (CIDR) devices to release progesterone on d 0. On d 14, the CIDRs were removed, and a 400 IU intramuscular injection of pregnant mare serum gonadotropin (Folligon) was administered to each ewe. The rams were introduced 24 h later. The next day, the gestation day count began starting on d 1 for all the ewes. The ewes underwent a trans-abdominal ultrasonography scan on gestation d 60 to confirm pregnancy and the number of fetuses. Weights were recorded one week before the beginning of the trial. The pregnant ewes’ weights ranged from 52 to 121 kg, and all ewes were in good health with body condition scores ranging from 2 to 3.5 at the beginning of the trial.

All ewes were housed in individual indoor pens and provided with a Se-deficient diet (< 0.05 mg Se/kg DM) and ad libitum water access. Ewes were individually fed twice daily at 8:00 and 15:30. After lambing, lambs stayed with their dams until weaning at postpartum d 50. On postpartum d 10, lambs were also given access to socialize in a common area with ad libitum creep feed and water to supplement their dam’s milk.

All ewes were randomly assigned to one of four treatment groups: 0 mg Se (control), 0.3 mg inorganic Se (0.3 mg iSe), 0.3 mg organic Se (0.3 mg oSe) and 0.6 mg organic Se (0.6 mg oSe). There were 27 ewes in the control group, 27 ewes in the 0.3 mg iSe group, 27 ewes in the 0.3 mg oSe and 29 ewes in the 0.6 mg oSe group. The daily Se dose was dissolved in 5 mL of a 1:1 solution of water and sucrose and administered orally using a 10-mL syringe; ewes in the control group received only the sucrose water as a sham control. Each ewe remained in the experiment receiving the same treatment through postpartum d 49 (PPD 49 or trial d 79). The Se requirements for sheep at different production stages can be seen in Table 1 [26].

**Table 1:** Se requirement calculations for ewes at different production stages [26]

Stage of production	Requirement calculation
Maintenance	0.00025 mg/kg × (BW/AC)
Growth	0.50 mg/kg × (LWG/AC)
Pregnancy (last third of gestation)	0.0025 mg/kg × (LBW/AC)
Lactation	0.14 mg/kg × (MY/AC)

*BW* Body weight (kg), *AC* Absorption coefficient (concentrates = 0.6 and forages = 0.31), *LWG* Live weight gain (kg/d), *LBW* Litter birth weight (kg), *MY* Milk yield (kg/d)

### Diet formulation and Se analyses

Both pre- and postpartum ewes received diets comprised of hay, a concentrate mix of whole barley and corn, and a protein pellet feed containing the mineral premix. Floradale Feed Mill Limited (Floradale, Ontario) provided the pelleted feed containing mineral premix for the trial, which excluded Se. The nutritional composition of the pelleted feed is recorded in Supplemental Table S1. Se levels in the 80:20 whole barley:corn grain were tested and considered negligible (0.032 µg/g). The first cut hay, sourced from Clinton, Ontario, was consistently below 0.05 µg/g Se, which is considered a negligible amount; animals regularly consuming less than 0.05 mg Se/kg DM have been found to be Se-deficient [15]. Feed Se levels were assessed by inductively coupled plasma mass spectrometry (ICP-MS) at the Animal Health Laboratory (AHL) in Guelph, Ontario. Prepartum ewes were provided with approximately 2.3 kg of hay per day as well as approximately 75 g of pelleted feed with 300 g of whole barley:corn mix in their last month of gestation. After lambing, their feed increased to 100–125 g of pelleted feed with 400–500 g of whole barley:corn grain. If a ewe’s body condition score was 2 or lower, additional feed was supplemented at the discretion of the barn staff.

### Preparation of OVA and LPS

The ovalbumin (OVA) antigen was sourced from Sigma-Aldrich (St. Louis, USA). A mixture of 12 mg of saponin (*Quillaja Saponaria*; Sigma-Aldrich, St. Louis, USA) and 12 mg OVA in 17.880 mL of phosphate-buffered saline (PBS) was prepared the day before use by vortexing in a 50-mL conical tube, then filter-sterilized with a 0.2-μm pore size filter and stored on ice until administered.

A solution containing *Escherichia coli* LPS endotoxin (O111:B4, 297-473-0, L2630, Sigma-Aldrich) was prepared the day before use from a 1 mg/mL frozen stock solution using PBS. Calculations were made based on the ewe’s body weight, so each animal received a 1 mL injection which included 400 ng LPS/kg body weight. The preparations were stored on ice until filter-sterilized before intravenous injection.

### Trial timeline

**Fig. 1:**
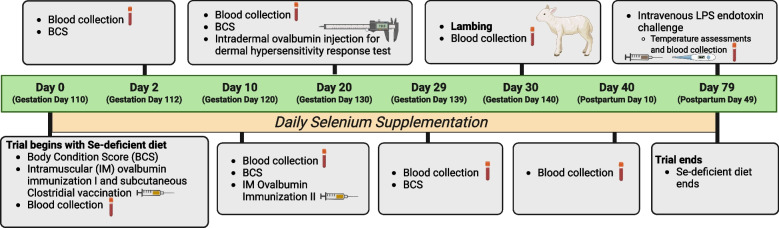
Animal trial timeline (Created in https://BioRender.com)

On trial d 0, all treatment ewes began consuming their Se-deficient diet and respective Se supplementation according to their treatment group, as shown in Fig. 1.

Each ewe received a TASVAX clostridial vaccination (Coopers^®^) on trial d 0 according to standard industry practice. On trial d 30 (GD 140), parturition was induced using a 4-mL intramuscular injection of 5 mg/mL dexamethasone (Vetquinol), after which lambing generally occurred within the following week. Blood samples were collected via venipuncture of the jugular vein using a 1″, 20-gauge needle, adapter, and plastic vacutainer serum tubes on trial d 0, 2, 10, 20, 29, 30, 40, and 79. Blood samples were allowed to clot at room temperature for a minimum of 30 min. Afterwards, blood was centrifuged at room temperature for 10 min at 2,000 × *g*. Sera was aliquoted into 1.5-mL sample tubes and frozen at −80 °C until used. Sera samples from trial d 0, 20, 30, 40 and 79 were sent to the University of Guelph AHL (Guelph, Ontario) to assess total serum Se concentrations using ICP-MS. Ewe body condition scores were evaluated on trial d 0, 2, 10, 20, and 29 using a five-point scale as described in Kennedy [27]. The trial ended on PPD 49.

### Assessment of antibody and dermal hypersensitivity response (DHR) to ovalbumin antigen

On trial d 0 and 10, the ewes received a 1-mL intramuscular injection of the OVA immunization preparation; each injection contained 0.67 mg of saponin and 0.67 mg of OVA. Serum collected on trial d 0, 10, and 20 was used to assess the ewe’s antibody response (IgG) to the OVA immunization.

The serum anti-OVA IgG levels were detected by an enzyme-linked immunoassay (ELISA) protocol. Briefly, a 96-well flat bottom clear polystyrene plate (Millipore, Darmstadt, Germany) was layered with 100 µL/well of a solution containing 14.4 mg OVA dissolved in 10 mL of coating buffer. The coating buffer was prepared by mixing 1.59 g sodium carbonate and 2.93 g sodium bicarbonate into 500 mL of deionized water. Before use, the coating buffer solution was filtered through a 0.22-µm bottle top filter in the fume hood, and the pH was adjusted to 9.6. The plate was sealed and incubated at 4 °C for 46–48 h. After this incubation period, the plate was washed 5 times with 0.05% PBS-Tween-20 (Thermo Scientific Chemicals, New Jersey, USA) wash buffer using a microplate washer from BioTek Instruments (Winooski, USA). The plate was blocked with 200 µL of Ultrablock provided by Bio-Rad (Oxford, UK) and incubated at room temperature for 1 h. The plate was washed as described above. Sera samples were thawed and diluted 1:200 in 0.05% PBS-Tween-20 wash buffer, then layered onto the antigen-coated 96-well plate. Standard curves were prepared using the pooled positive (trial d 20) and negative control (trial d 0) samples and included on each plate. Each well received 100 µL of sample in duplicate before a 2-h incubation period at room temperature. After this, the plate was washed again as described above. Rabbit anti-sheep (H + L)-HRP-conjugated IgG antibody (Bio-Rad Laboratories, USA) was diluted in wash buffer at 1:1,000, and 100 µL of the solution was added to each well. After a 1-h room temperature incubation period, 80 µL of 3,3′,5,5′-tetramethylbenzidine (TMB) (Sigma-Aldrich, St. Louis, USA) was added to each well. After 45 min of room temperature incubation in the dark, the optical density for each well was measured at 450 nm using a Cytation 5 imaging reader (BioTek Instruments, Winooski, USA). The inter-assay coefficient of variation (CV) was 8.4%. The intra-assay CV was consistently below 10%.

The ewes were subjected to a dermal hypersensitivity response test using the OVA antigen on trial d 20 (GD 130). The right or left side of the lower neck regions of the ewes were shaved down to the skin. On this shaved region, two sites were marked, one for the 0.5 mL OVA antigen intradermal injection and the other for the 0.5 mL PBS, which served as the sham negative control. Each OVA injection contained 0.34 mg OVA and 0.34 mg saponin. 0.5 mL of the OVA or PBS preparations were intradermally injected at the appropriate site with a 28G needle and 2 mL syringe. At both sites, skinfold thickness was measured in three repetitions at 0, 6, 24, and 48 h post intradermal OVA challenge using a Harpenden skinfold caliper (Baty International, UK). The mean of the three measurements was calculated for each time point. To calculate the increase in skinfold thickness, the ratio of the mean skinfold thickness measured at timepoints 6, 24, or 48 h post intradermal injection over the mean skinfold thickness measured at timepoint 0 was used. All the ratios for each time point were averaged within each treatment group per block for statistical analysis.

### Assessment of acute-phase protein and metabolite response to LPS challenge

A LPS challenge was performed the day before weaning on trial d 79 (PPD 49). Ewes were intravenously injected with 1 mL of 400 ng/kg LPS solution in the jugular vein at h 0, as described in Shandilya et al. [28]. The ewes’ rectal temperatures were recorded hourly until 4 h post injection using a standard digital thermometer. Blood was collected at time points 0, 4 and 24 h post-LPS injection to obtain serum. Blood samples were processed and stored using the aforementioned protocol. Sera samples from time points 0, 4 and 24 were sent to Animal Health Laboratories (Guelph, Canada) for the full biochemical panel profile analyses, which included serum ions, protein, liver enzymes and select metabolites, as seen in Supplemental Table S2. Only the parameters that had a significant time or treatment effect (*P* < 0.05) were included in the results.

Sera samples from hourly time points 0 and 4 were thawed and used for analysis of GPx activity using a Glutathione Peroxidase Assay Kit from Cayman Chemical (Ann Arbor, USA) to assess antioxidant capacity. Briefly, background, positive control, and sample wells were filled with assay buffer, co-substrate, and nicotinamide adenine dinucleotide phosphate (NADPH). The positive control wells had 20 µL of diluted bovine GPx provided in the kit additionally added. The sample wells had 20 µL of serum aliquoted to each in duplicate. Cumene hydroperoxide (20 µL) was added to all the wells to initiate the GPx reaction. After shaking the plate for 10 s, the optical density was read once per min for 5 min at 340 nm using a Cytation 5 plate reader (BioTek). The GPx activity was calculated based on the rate of change in absorbance; a decrease in absorbance directly correlates with a reduction in NADPH content in the samples, indicating that NADPH was being used to reduce the glutathione disulfide (GSSG) product back to glutathione (GSH).

Sera samples from time points 0 and 4 were also thawed to assess cortisol concentrations using the DetectX^®^ Cortisol Enzyme Immunoassay Kit provided by Arbor Assays (Ann Arbor, USA) to assess the stress response. Briefly, samples or kit standards were added to the goat anti-mouse IgG coated plate in duplicate. Next, assay buffer, cortisol conjugate, and cortisol antibody were added to the wells as detailed in the manufacturer’s instructions. The plate was sealed and left to shake at room temperature for 1 h at 700–900 r/min. The plate was aspirated and washed 4 times with wash buffer. TMB substrate was added to each well, and the plate was incubated for 30 min in the dark at room temperature. After the incubation, stop solution was added and the optical density was read at 450 nm using a Cytation 5 plate reader (BioTek). Cortisol concentrations were determined using a 4PLC software provided by MyAssays^®^. The intra-assay CV was consistently below 10%.

Sera samples from time points 0 and 4 were also sent to Eve technologies (Calgary, Canada) for cytokine multiplexing using the Luminex™ 200™ instrument system based on a flow cytometry platform to detect IFN-γ, interleukin (IL)-6, IL-10, and interferon gamma-induced protein 10 (IP-10). These cytokines were selected based on their involvement in the innate inflammatory processes.

### Statistical analyses

All statistical analyses were performed using R version 4.3.2 by R Core Team (2023). The study followed a randomized complete block design with repeated measures, where the “ewe” was considered the experimental unit. The statistical model was *Y*_*ijk*_ = *µ* + *Treatment*_*i*_ + *Block*_*j*_ + *Time*_*k*_ + *Treatment × Time*_*ik*_ + *e*_*ijk*_, where *Y* = dependent variable, *µ* = overall mean, *Treatment*_*i*_ = fixed effect of selenium treatment, *Block*_*j*_ = random effect of block, *Time*_*k*_ = fixed effect of time, *Treatment × Time*_*ik*_ = interaction between treatment and time, and* e*_*ijk*_ = residual error.

A linear mixed effect model (using the lmer() function—lme4) was used to test fixed and random effects. To account for repeated measures over time, different residual covariance structures were tested using the gls() and lme() functions—nlme, and the best-fitting model was selected based on the lowest Akaike Information Criterion (AIC). Least square means were compared pairwise using the adjusted Tukey for multiple comparisons (emmeans function). For the serum Se and anti-OVA antibody analysis, the data collected on d 0 of the trial, when no Se treatments had been applied, were included as covariates to account for baseline differences among the ewes. Residual analyses were performed to evaluate model assumptions. Homogeneity of the residual variance across the time and treatment levels were evaluated visually using boxplots. Normality of residuals was also assessed using the Shapiro–Wilk test and quantile–quantile plot. When violations of ANOVA assumptions were detected, appropriate transformations or generalized linear models with suitable link functions were performed. All results were presented as the least squares means (LSM) ± standard error of the mean (SEM). Statistical significance was determined at *P* ≤ 0.05.

## Results

### Serum Se concentration

**Fig. 2:**
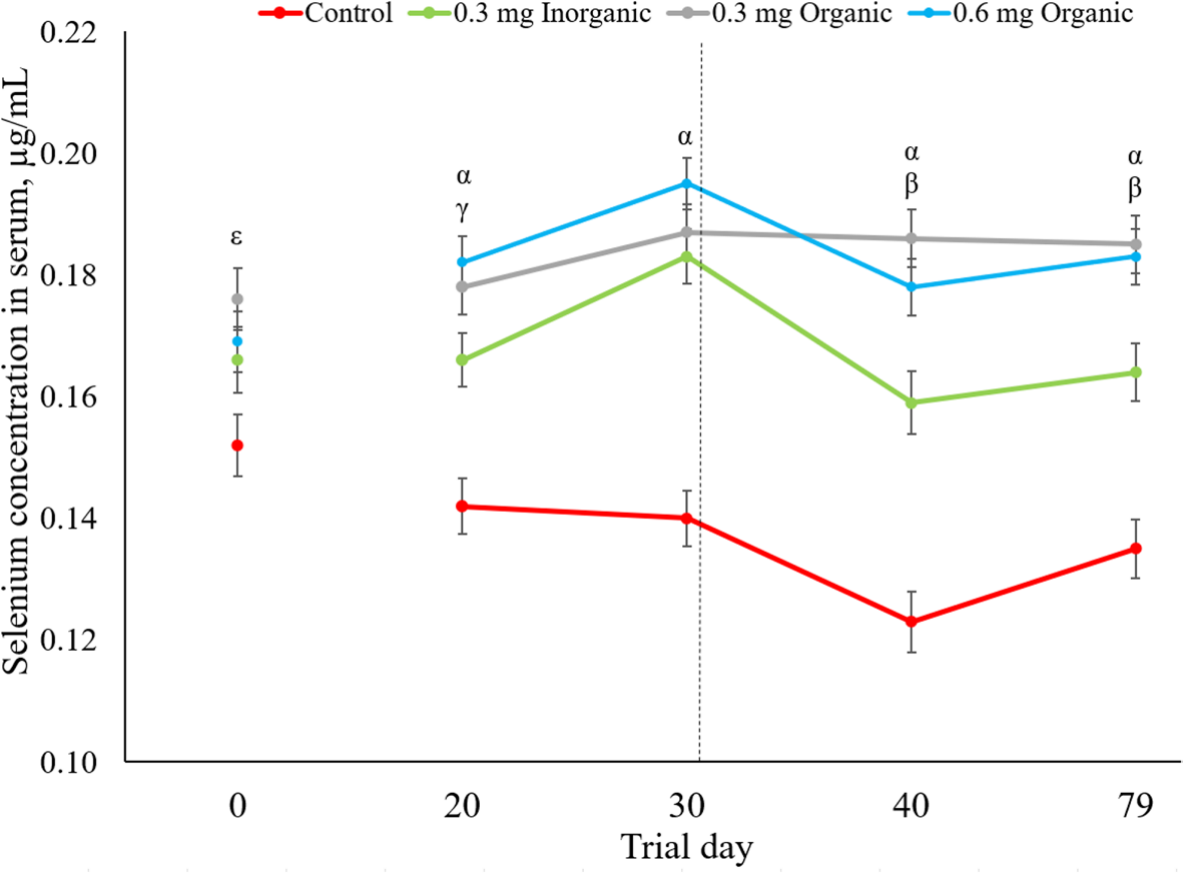
Ewe serum selenium concentrations on trial d 0, 20, 30, 40 and 79. Data are presented as least squares mean ± standard error of the mean. Significant differences (*P* < 0.05) across treatments are indicated by letters where α = significance between the Se treatments and control group, β = significance between both oSe and 0.3 mg iSe groups, γ = significance between 0.6 mg oSe and 0.3 mg iSe group, ε = significance between 0.3 mg oSe group and control group

The results of serum Se concentrations over the trial period are displayed graphically in Fig. 2. Both treatment and day were significant (*P* < 0.001), and the interaction between treatment and day was also significant (*P* = 0.001).

On d 0 of the trial, the ewes in the 0.3 mg organic treatment group had a higher concentration of serum Se than the control group (*P* = 0.003), while there was a tendency for the 0.6 mg group to have a higher concentration than the control group (*P* = 0.058). The differences in basal serum Se concentrations were attributed to natural Se status variations in the flock, as the animals were randomly assigned to each treatment group. Serum Se concentrations on d 0 were set as a covariate for statistical analysis for the subsequent trial days.

On trial d 20, 30, 40 and 79, all Se supplemented groups had greater concentrations of serum Se than the control group (*P* < 0.050). On trial d 20, 40 and 79, the ewes in the 0.6 mg oSe group had higher serum Se concentrations than the 0.3 mg iSe group, while on trial d 40 and 79, the 0.3 mg oSe group had higher serum Se concentrations than the 0.3 mg iSe group (*P* < 0.050). There were no differences in Se serum concentrations between the ewes in the 0.3 mg and 0.6 mg oSe groups at any point during the trial. For the ewes of the 0.3 mg iSe and 0.6 mg oSe groups, serum concentrations on trial d 30 were higher than trial d 20, 40, and 79 (*P* < 0.031). For the control group, serum Se concentrations on trial d 40 were lower than trial d 20 and 30 (*P* < 0.002), while tending to be lower than d 79 (*P* < 0.054).

### OVA-specific adaptive immune response

**Fig. 3:**
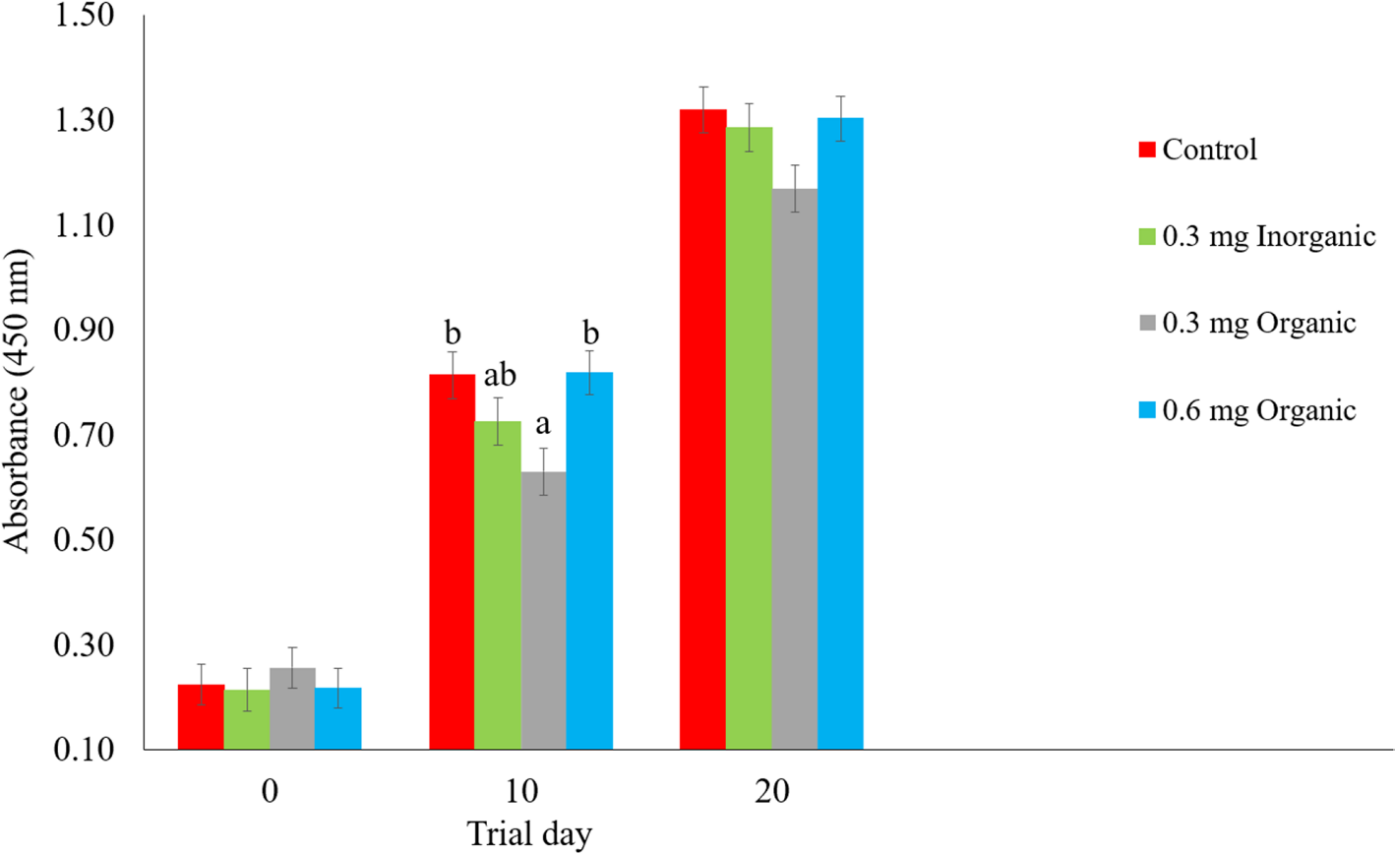
Effect of selenium on serum anti-ovalbumin IgG levels on trial d 0, 10, and 20. Data are presented as least squares mean ± standard error of the mean

Upon assessing the effect of Se on anti-OVA IgG levels over time, treatment was significant (*P* = 0.008), and time was significant (*P* < 0.001). The interaction between treatment and time was not significant (*P* = 0.778). For all treatment groups, there was an increase in the relative serum anti-OVA IgG levels on trial d 20 compared to d 10, indicating they responded as expected to the antigen (Fig. 3). On trial d 10, the 0.3 mg oSe group had lower anti-OVA IgG levels in comparison to the 0.6 mg oSe and control groups (*P* < 0.021). The latter two groups did not differ. In addition, 0.3 mg iSe had an intermediate value and did not differ from any other group. No differences between treatment groups were observed on d 20. However, there was a tendency for the 0.3 mg oSe group to have lower IgG levels than the control group (*P* = 0.082).

For the dermal hypersensitivity response to OVA, the percentage increase of skinfold thickness in response to OVA antigen was compared to that induced by PBS within each treatment group (data not presented). In comparison to the dermal response to PBS, the dermal response to OVA was significantly greater at all time points for all treatment groups (*P* < 0.001). The average fold change between the percent skinfold thickness increase from the saline site to the OVA site was 41.53 ± 0.33. This demonstrates that the ewes did have an immunological response to the OVA. However, there were no statistically significant differences (*P* > 0.050) induced by time or treatment, as shown in Table 2.

**Table 2:** Effect of selenium on dermal hypersensitivity response to ovalbumin at 6, 24, and 48 h post intradermal injection

Hours post-intradermal ovalbumin injection	Treatment				*P*-value		
	Control	0.3 mg inorganic selenium	0.3 mg organic selenium	0.6 mg organic selenium	Treatment	Time	Treatment × Time
					0.632	0.424	0.918
6	1.40 ± 0.0557	1.44 ± 0.0557	1.47 ± 0.0557	1.47 ± 0.0557			
24	1.41 ± 0.0557	1.43 ± 0.0557	1.53 ± 0.0557	1.47 ± 0.0557			
48	1.40 ± 0.0557	1.42 ± 0.0557	1.47 ± 0.0557	1.44 ± 0.0557			

Data are presented as the least squares mean ratio (skinfold thickness in mm at the time point/original skinfold thickness in mm) ± standard error of the mean

### Acute-phase response to LPS challenge

There was a significant decrease in serum GPx activity between 0 to 4 h post-LPS injection, which was driven chiefly by the 0.3 mg iSe group (Table 3). However, no treatment effect was observed.

**Table 3:** Effect of selenium on serum glutathione peroxidase (GPx) activity (nmol/min/mL) 0 and 4 h post lipopolysaccharide endotoxin (400 ng/kg) intravenous injection

Hours post-LPS injection	Treatment				*P*-value		
	Control	0.3 mg inorganic selenium	0.3 mg organic selenium	0.6 mg organic selenium	Treatment	Time	Treatment × Time
					0.383	0.026	0.319
0	253 ± 25.1	332^α^ ± 24.0	275 ± 25.1	290 ± 24.0			
4	241 ± 25.1	270^β^ ± 24.0	272 ± 25.1	263 ± 24.0			

Data are presented as least squares mean ± standard error of the mean. Differing Greek letter superscripts indicate statistically significant differences (*P* ≤ 0.050) within the same column

Across all treatment groups, there was an increase in serum cytokine concentrations from 0 to 4 h post-LPS injection (*P* < 0.050), as depicted in Table 4. However, there were no treatment-induced differences.

**Table 4:** Effect of selenium supplementation on serum cytokine concentrations (pg/mL) 0 and 4 h post lipopolysaccharide endotoxin (400 ng/kg) intravenous injection

Cytokine	Hours post-LPS injection	Treatment				*P*-value		
		Control	0.3 mg inorganic selenium	0.3 mg organic selenium	0.6 mg organic selenium	Treatment	Time	Treatment × Time
IFN-γ						0.585	< 0.001	0.805
	0	5.40 ± 5.37	9.30 ± 5.37	4.30 ± 5.14	7.09 ± 4.94			
	4	15.52 ± 5.37	27.37 ± 5.37	19.81 ± 5.14	23.55 ± 4.94			
IL-6						0.936	< 0.001	0.899
	0	176 ± 2,359	173 ± 2,258	271 ± 2,258	207 ± 2,170			
	4	6,230 ± 2,359	1,132 ± 2,258	9,980 ± 2,258	8,939 ± 2,170			
IL-10						0.273	< 0.001	0.941
	0	546 ± 162	332 ± 162	341 ± 162	468 ± 156			
	4	1,308 ± 162	1,039 ± 162	1,184 ± 162	1,511 ± 156			
IP-10						0.733	< 0.001	0.370
	0	407 ± 155	420 ± 155	418 ± 155	392 ± 146			
	4	1,685 ± 155	1,648 ± 155	1,399 ± 155	1,468 ± 146			

Data are presented as least squares mean ± standard error of the mean

*IFN-γ* Interferon gamma, *IL-6* Interleukin 6, *IL-10* Interleukin 10, *IP-10* Interferon gamma induced protein 10

**Fig. 4:**
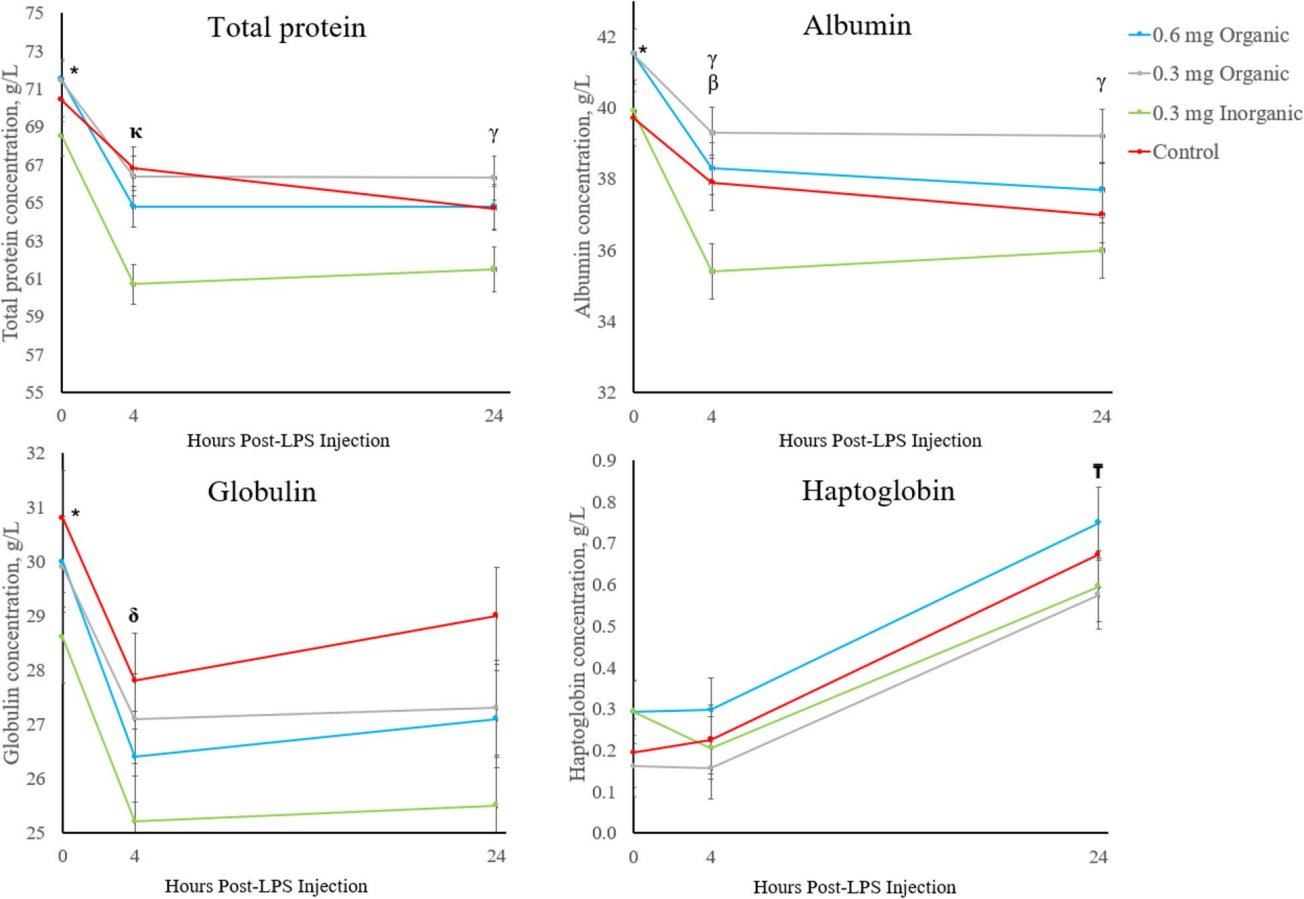
Effect of Se supplementation on serum haptoglobin, total protein, globulin, and albumin concentrations 0, 4 and 24 h post-lipopolysaccharide endotoxin (400 ng/kg) intravenous injection. Data are presented as least squares mean ± standard error of the mean. Significant differences (*P* < 0.050) across treatments are indicated by symbols where **κ** = significance between 0.3 mg iSe and all other groups, **γ** = significance between 0.3 mg oSe and 0.3 mg iSe, **β** = significance between 0.6 mg oSe and 0.3 mg iSe, **δ** = significance between control and 0.3 mg iSe, **₸** = significance between time point 24 and all other time points for all treatment groups, * = significance between time point 0 and all other time points for all treatment groups

For the biochemical analysis of serum total protein, albumin, globulin and haptoglobin concentrations, the *P*-values for treatment, time, and treatment × time are presented in Table 5. Hour 0 was higher than all other time points post-LPS injection for serum total protein, albumin, and globulin concentrations across all treatment groups (*P* < 0.050), as displayed in Fig. 4. For serum haptoglobin, 24 h concentrations were significantly higher than all other time points across all treatments (*P* < 0.050).

**Table 5:** *P*-values of interest for serum total protein, albumin, globulin, and haptoglobin

Parameter	*P*-value		
	Treatment	Time	Treatment × Time
Total Protein	0.002	< 0.001	0.200
Albumin	0.014	< 0.001	0.028
Globulin	0.114	< 0.001	0.534
Haptoglobin	0.325	< 0.001	0.945

Regarding treatment differences, the total serum protein concentration was lower in the 0.3 mg iSe group at h 4 in comparison to other groups (*P* < 0.040), while the 0.3 mg oSe group was higher than the 0.3 mg iSe group at h 24 (*P* = 0.020). For serum albumin concentrations, both oSe groups were higher than the 0.3 mg iSe group at h 4 (*P* < 0.030), while there was a tendency for the control group to be higher than the 0.3 mg iSe group at h 4 (*P* = 0.096). At h 24, the 0.3 mg oSe group was higher than the 0.3 mg iSe group (*P* = 0.020). For the serum globulin concentration, the control group was higher than the 0.3 mg iSe group at h 4 (*P* = 0.015).

Table 6 presents the results from the serum biochemical analyses h 0 and 4 post-LPS endotoxin intravenous injection. The following biochemical parameters had differences (*P* < 0.050) in concentration induced by time: A:G ratio, urea, creatinine, glucose, cholesterol, ALP, GGT, BHBA, NEFA, calculated osmolality, Ca, P, Mg, Na, K, Cl, anion gap, and N:K ratio. The following biochemical parameters had statistically significant (*P* < 0.050) treatment-induced changes at one or more of the time points: calculated osmolality, Na, K, Cl, Na:K ratio.

**Table 6:** Effect of Se supplementation on ewe serum biochemical parameters at h 0, 4, and 24 post lipopolysaccharide endotoxin intravenous injection

Parameter	Hours post-LPS injection	Treatment group				*P*-value		
		Control	0.3 mg inorganic selenium	0.3 mg organic selenium	0.6 mg organic selenium	Treatment	Time	Treatment × Time
Albumin:Globulin (A:G) ratio						0.284	0.009	0.436
	0	1.29 ± 0.0538	1.41 ± 0.0509	1.4^α^ ± 0.0507	1.4^α^ ± 0.0509			
	4	1.32 ± 0.0538	1.42 ± 0.0509	1.48^β^ ± 0.0507	1.46^β^ ± 0.0509			
	24	1.34 ± 0.0538	1.42 ± 0.0528	1.45^αβ^ ± 0.0523	1.41^αβ^ ± 0.0528			
Urea, mmol/L						0.247	< 0.001	0.589
	0	6.44 ± 0.359	6.46^αβ^± 0.337	7.21 ± 0.334	6.88^α^ ± 0.337			
	4	7.08 ± 0.359	6.97^α^ ± 0.337	7.72 ± 0.334	7.5^β^ ± 0.337			
	24	6.96 ± 0.359	6.22^β^ ± 0.358	7.33 ± 0.353	6.93^αβ^ ± 0.358			
Creatinine, µmol/L						0.962	0.004	0.119
	0	69.6^α^± 5.04	70.8 ± 4.75	69.6 ± 4.72	71.9 ± 4.75			
	4	75.5^αβ^ ± 5.04	77.4 ± 4.75	75.8 ± 4.72	78.8 ± 4.75			
	24	81.8^β^ ± 5.04	71.3 ± 5.00	71.9 ± 4.93	72.9 ± 5.00			
Glucose, mmol/L						0.257	< 0.001	0.961
	0	3.5^α^ ± 0.19	3.52^αβ^ ± 0.174	3.57^α^ ± 0.17	3.78^α^ ± 0.174			
	4	4.14^β^ ± 0.19	3.9^α^ ± 0.174	4.17^β^ ± 0.17	4.32^β^ ± 0.174			
	24	3.13^α^ ± 0.19	3.15^β^ ± 0.197	3.36^α^ ± 0.191	3.49^α^ ± 0.197			
Cholesterol, mmol/L						0.490	< 0.001	0.484
	0	3.50^α^± 0.190	3.52^αβ^ ± 0.174	3.57^α^ ± 0.170	3.78^α^ ± 0.174			
	4	4.14^β^ ± 0.190	3.90^α^ ± 0.174	4.17^β^ ± 0.170	4.32^β^ ± 0.174			
	24	3.13^α^ ± 0.190	3.15^β^ ± 0.197	3.36^α^ ± 0.191	3.49^α^ ± 0.197			
Alkaline phosphatase (ALP), U/L						0.701	0.012	0.781
	0	109.9 ± 14.3	104.9 ± 13.5	103.5 ± 13.4	96.7^α^ ± 13.5			
	4	107.9 ± 14.3	95.9 ± 13.5	94.2 ± 13.4	78.8^β^ ± 13.5			
	24	117.4 ± 14.3	101.6 ± 14.0	100.1 ± 13.8	94.9^αβ^ ± 14			
Gamma glutamyl transferase (GGT), U/L						0.397	< 0.001	0.437
	0	75.8 ± 7.77	83.4^α^ ± 7.42	66 ± 7.41	69.4^α^ ± 7.42			
	4	74.3 ± 7.77	76.6^β^ ± 7.42	62.2 ± 7.41	65.1^αβ^± 7.42			
	24	74.7 ± 7.77	75.7^β^ ± 7.49	61 ± 7.47	62.4^β^ ± 7.49			
Beta hydroxybutyrate (BHBA), µmol/L						0.820	0.021	0.717
	0	480 ± 43.5	484 ± 40.4	477 ± 39.8	479^α^ ± 40.4			
	4	428 ± 43.5	444 ± 40.4	457 ± 39.8	367^β^ ± 40.4			
	24	455 ± 43.5	487 ± 44.5	454 ± 43.4	441^αβ^ ± 44.5			
Non-esterified fatty acids (NEFA), mmol/L						0.853	0.001	0.456
	0	0.185 ± 0.042	0.275^α^ ± 0.0385	0.224 ± 0.0374	0.262^α^ ± 0.0385			
	4	0.128 ± 0.042	0.112^β^ ± 0.0385	0.157 ± 0.0374	0.118^β^ ± 0.0385			
	24	0.182 ± 0.042	0.188^αβ^ ± 0.044	0.201 ± 0.0424	0.124^β^ ± 0.044			
Calculated osmolality, mmol/L						0.016	< 0.001	0.597
	0	291 ± 2.21	296^α^ ± 2.04	298^αβ^ ± 2	296 2.04			
	4	291^a^ ± 2.21	294^αβ, ab^ ± 2.04	300^α,b^ ± 2	296^ab^2.04			
	24	288 ± 2.21	289^β^ ± 2.28	293^β^ ± 2.22	291 ± 2.28			
Calcium (Ca), mmol/L						0.138	< 0.001	0.845
	0	2.65^α^ ± 0.0519	2.71^α^ ± 0.048	2.76^α^ ± 0.0471	2.69^α^ ± 0.048			
	4	2.35^β^ ± 0.0519	2.33^β^ ± 0.048	2.47^β^ ± 0.0471	2.36^β^ ± 0.048			
	24	2.38^β^ ± 0.0519	2.39^β^ ± 0.0533	2.5^β^ ± 0.0519	2.39^β^ ± 0.0533			
Phosphorous (P), mmol/L						0.323	< 0.001	0.409
	0	1.419^α^ ± 0.0953	1.406^α^ ± 0.0876	1.461^α^ ± 0.0855	1.58^α^ ± 0.0876			
	4	0.902^β^ ± 0.0953	0.837^β^ ± 0.0876	0.842^β^ ± 0.0855	0.873^β^ ± 0.0876			
	24	1.428^α^ ± 0.0953	1.108^γ^ ± 0.0992	1.285^α^ ± 0.0959	1.433^α^ ± 0.0992			
Magnesium (Mg), mmol/L						0.912	< 0.001	0.393
	0	1.066^α^ ± 0.0309	1.117^α^ ± 0.0287	1.086^α^ ± 0.0283	1.075^α^ ± 0.0287			
	4	1.045^αβ^ ± 0.0309	1.067^α^ ± 0.0287	1.063^α^ ± 0.0283	1.05^α^ ± 0.0287			
	24	0.983^β^ ± 0.0309	0.932^β^ ± 0.0316	0.944^β^ ± 0.0308	0.928^β^ ± 0.0316			
Sodium (Na), mmol/L						0.041	< 0.001	0.564
	0	146 ± 1.11	149^α^ ± 1.03	149^αβ^ ± 1.01	148 ± 1.03			
	4	146^a^ ± 1.11	147^αβ, ab^ ± 1.03	151^α, b^ ± 1.01	148^ab^ ± 1.03			
	24	145 ± 1.11	146^β^ ± 1.15	147^β^ ± 1.12	146 ± 1.15			
Potassium (K), mmol/L						0.021	< 0.001	0.254
	0	4.71^α^ ± 0.137	5.01 ± 0.126	4.91^α^ ± 0.123	4.88^α^ ± 0.126			
	4	4.03^αβ, a^ ± 0.137	4.76^b^ ± 0.126	4.56^β, b^ ± 0.123	4.48^β, ab^ ± 0.126			
	24	4.52^α^ ± 0.137	4.68 ± 0.143	4.78^αβ^ ± 0.138	4.82^αβ^ ± 0.143			
Chloride, mmol/L						0.009	< 0.001	0.121
	0	106 ± 0.854	109^α^ ± 0.784	108^α^ ± 0.766	107^α^ ± 0.784			
	4	107^a^ ± 0.854	109^α, ab^ ± 0.784	111^β, b^ ± 0.766	110^β, ab^ ± 0.784			
	24	104 ± 0.854	106^β^ ± 0.889	106^α^ ± 0.859	106^α^ ± 0.889			
Anion gap, mmol/L						0.330	< 0.001	0.201
	0	24.5 ± 0.92	26.5^α^ ± 0.858	26.9^α^ ± 0.848	27.3^α^ ± 0.858			
	4	22.8 ± 0.92	22.8^β^ ± 0.858	23.5^β^ ± 0.848	24.4^β^ ± 0.858			
	24	23.8 ± 0.92	23.1^β^ ± 0.931	25.1^αβ^ ± 0.912	24.2^β^ ± 0.931			
Na:K ratio						0.046	< 0.001	0.0454
	0	31.2^α^ ± 0.9	30 ± 0.826	30.7^α^ ± 0.806	30.7^α^ ± 0.826			
	4	36.7^β, a^ ± 0.9	31.2^b^ ± 0.826	33.2^β, b^ ± 0.806	33.3^β, b^ ± 0.826			
	24	32.3^α^ ± 0.9	31.6 ± 0.938	31^αβ^ ± 0.906	30.6^α^ ± 0.938			

Data are presented as least squares mean ± standard error of the mean. Means followed with different superscript letters differ significantly (*P* ≤ 0.050). Means followed with differing Greek letter superscripts indicate statistically significant differences (*P* ≤ 0.050) between time points within the same treatment group

As displayed in Table 7, there was an increase in serum cortisol concentrations between h 0 and 4 across all treatment groups (*P* < 0.001). However, there were no significant treatment-induced effects at any time point (*P* > 0.265).

**Table 7:** Effect of selenium on serum cortisol concentrations (ng/mL) at h 0 and 4 post lipopolysaccharide endotoxin (400 ng/kg) intravenous injection

Hours post-LPS injection	Treatment				*P*-value		
	Control	0.3 mg inorganic selenium	0.3 mg organic selenium	0.6 mg organic selenium	Treatment	Time	Treatment × Time
					0.3502	< 0.0001	0.4470
0	13.672 ± 7.200	17.730 ± 7.569	21.838 ± 7.200	15.904 ± 7.200			
4	102.450 ± 7.200	77.949 ± 7.569	108.048 ± 7.200	97.255 ± 7.200			

Data are presented as least squares mean ± standard error of the mean

Ewe rectal temperatures increased across time points in all treatment groups (*P* < 0.001), as shown in Table 8. At h 0, the 0.3 mg iSe group had a higher rectal temperature than the control group (*P* = 0.007), but no other treatment effects were observed at any other time point (*P* > 0.130).

**Table 8:** Effect of selenium on ewe rectal temperatures (ºC) in h 0, 1, 2, 3, and 4 post lipopolysaccharide endotoxin (400 ng/kg) intravenous injection

Hours post-LPS injection	Treatment				*P*-value		
	Control	0.3 mg inorganic selenium	0.3 mg organic selenium	0.6 mg organic selenium	Treatment	Time	Treatment × Time
					0.0007	< 0.0001	0.0089
0	38.7^α, a^ ± 0.108	39.2^α, b^ ± 0.111	39.1^α, ab^ ± 0.111	39.1^α, ab^ ± 0.106			
1	39.5^β^ ± 0.110	39.5^β^ ± 0.111	39.4^β^ ± 0.111	39.8^β^ ± 0.106			
2	39.7^β^ ± 0.110	39.8^γ^ ± 0.110	39.7^γ^ ± 0.111	39.9^β^ ± 0.106			
3	40.4^γ^ ± 0.111	40.5^δ^ ± 0.110	40.4^δ^ ± 0.111	40.7^γ^ ± 0.106			
4	40.7^γ^ ± 0.111	41.0^ε^ ± 0.108	40.8^ε^ ± 0.111	40.8^γ^ ± 0.106			

Data are presented as least squares mean ± standard error of the mean. Means followed with different superscript letters differ significantly (*P* ≤ 0.05) within rows. Means followed with differing Greek letter superscripts indicate statistically significant differences (*P* ≤ 0.05) within columns

## Discussion

Ensuring an adequate Se status is one essential way to promote the health of animals. With rising concerns of drug resistance and known geographical areas of soil Se deficiencies, supplementing animals to boost their resilience against diseases and pathogens is critical [29]. Since Se has numerous mechanisms of action to promote the function of the immune system [1, 3, 29], the immunomodulatory impact of Se supplementation during late gestation and lactation is worth investigating to give producers more tools to improve ovine immune health.

As expected, the Se supplemented groups had higher Se serum concentrations than the Se-deficient control group on every trial day beyond d 0. However, it was interesting that the control ewes receiving the Se-deficient diet for 79 d never showed any clinical signs of Se deficiency and did not have critically low serum Se levels (< 0.060 µg/mL) [30], suggesting that these ewes may have accessed Se reserves in their muscle tissues to meet their Se requirements [31, 32]. When comparing the 0.3 mg oSe and the iSe group, the 0.3 mg oSe treatment maintained the ewe serum Se concentrations higher than those of the 0.3 mg iSe group only on trial d 40 and 79, showing that the benefits of oSe supplementation in significantly raising serum Se status may take a period of adaptation to reach a steady state, yet oSe may be more effective in maintaining higher serum Se levels in the long term. These results are in agreement with previous research showing that oSe supplementation significantly enhanced serum Se concentrations and is more bioavailable to the animal for utilization in the body in comparison to iSe sources due to the degradation of iSe into elemental Se in the rumen, lack of storage potential and higher rates of urinary losses [12, 22, 32–34]. However, given that there were no differences in serum Se concentrations when comparing the 0.3 mg and 0.6 mg oSe group, supplying additional oSe did not appear to benefit the ewe’s overall Se status. Nonetheless, previous research has shown that far higher supranutritional doses of oSe improved animal Se status; moreover, no homeostatic control over absorption exists to limit Se status [2, 11, 35, 36]. Thus, a limitation in absorption is likely not why the 0.6 mg oSe group did not differ in Se serum concentrations to 0.3 mg oSe group. One possibility is that the extra 0.3 mg Se in the 0.6 mg group was partitioned into the ewe’s or developing lamb’s muscle through SeMet’s replacement of methionine in general protein synthesis or excreted in the milk.

In terms of Se’s role in immune function, leukocyte functions are dependent on normal functioning of various selenoproteins, which help to regulate cellular processes, including differentiation, activation, proliferation, oxidative bursts, signaling, phagocytosis, protein folding, antioxidant activities, and cytokine secretion [3]. For example, selenoprotein K is needed to allow the flux of Ca^2+^ into cells for successful activation of macrophages, neutrophils, and T cells [3]. Critical antioxidant selenoproteins, such as GPx1, also play a role in controlling oxidative burst reactions and regulating H_2_O_2_ production [3]. However, despite changes in serum Se status, no major immunological differences amongst the treatment groups were detected.

First, when assessing the prepartum adaptive immune response, there were only differences in serum anti-OVA antibody levels on d 10, yet the trend was still observed on d 20. Studies in other species have found that oSe supplementation enhanced antibody production; however, the immunization protocol was not initiated until after a period Se-treatment acclimatization, whereas, the ewes in the present study were immunized on trial d 0 when they first started receiving their Se supplement [19, 37–39]. For future trials, providing the Se supplement earlier in gestation may be beneficial so that their Se status has adequate time to change before exposing them to a novel antigen. There were also no treatment or time differences in the DHR to OVA. Previous researchers pointed out that the DHR to OVA may be a type III hypersensitivity response caused by antigen-antibody complexes, neutrophils, and mast cells accumulating at the injection site [40]. Since our study did not include skin biopsies of the injection site to assess what inflammatory cells were present, it is impossible to determine exactly what type of immune response occurred. This may be a valuable addition to future studies. Past murine research indicates that Se does have an attenuating effect on dermal immune responses by reducing inflammatory cell infiltration and IL-4 expression [41]. Likewise, a porcine study found that piglets born to Se supplemented sows had attenuated skinfold edema in response to the intradermal OVA injection in comparison to non-supplemented sows [37]. However, a randomized controlled trial in humans found that participants taking a high Se yeast supplement maintained stronger DHRs to selected antigens in comparison to a low Se yeast supplement control group, showing the need of further exploration of this topic as the effect of Se may depend on a combination of the antigen, adjuvant or type of dermal hypersensitivity response [42].

In our assessment of the acute-phase response, there were also no treatment associated differences in the total serum GPx activity during the LPS challenge on trial d 79. Interestingly, on this same day, there was a difference in Se serum concentrations when comparing the oSe groups, 0.3 mg iSe group and control group. This finding may indicate that the concentrations of serum Se needed to maximize GPx activity had been surpassed in all treatment groups, denoting that supplementing with Se did not confer any biological antioxidant benefits for serum GPx activity [43]. Another ovine study assessing different Se supplementation rates (0.15, 0.30, and 0.45 mg/kg diet) did not find a significant effect on GPx activity either, even after 40 d of the experimental diet [19]. Alternatively, one study showed that the sheep receiving 0.86 mg Se per day over 3 months had enhanced GPx activity in their blood, liver, kidney cortex, duodenum, and ileum in comparison to the non-supplemented control group; though there was no difference in Se yeast supplementation compared to sodium selenite [44]. Nonetheless, we did not assess the GPx activity or expression levels in the spleen, thymus, intestine, erythrocytes, or lymphocytes, as has been done in other studies to serve as biomarkers for Se status [23, 43, 44]. On the other hand, there was a decrease in GPx activity between time 0 and 4 driven by the 0.3 mg inorganic treatment group. This finding suggests that the LPS challenge may have induced greater oxidative stress that needed to be reverted by the sera GPX activity in ewes from the 0.3 mg inorganic group. It is surprising that the other treatment groups did not have a drop in GPx activity between h 0 and 4 post-LPS injection, as studies have shown that LPS challenges reduce antioxidant activity in the blood [45, 46]. However, since the ewe’s diets were not depleted of vitamin E, another dietary antioxidant, the presence of vitamin E and other antioxidant enzymes, such as catalase and superoxide dismutase, may have played a more critical role in maintaining oxidative balance during the LPS challenge rather than GPx.

As expected, our study results found that the LPS endotoxin injection successfully stimulated the production of the major cytokine IL-6, agreeing with previous literature [47, 48]. IL-6 plays an important role in regulating inflammation and the adaptive immune response; it also promotes the production of the anti-inflammatory IL-10 cytokine, which was induced by our LPS challenge and in previous studies [49, 50]. IL-10 is secreted by T-helper (Th)2 cells and M2 macrophages and suppresses T cell, NK cell, and macrophage activity, while reducing overall inflammation [49]. IFN-γ concentrations significantly increased between h 0 and h 4 post-LPS injection, which is consistent with findings from other studies [48, 50]. IFN-γ is synthesized by CD4^+^ Th1, CD8^+^ T cells, and NK cells; this cytokine supports the cell-mediated immune response and affects the activities of other T cells, NK cells and macrophages [49]. IP-10, also known as CXC ligand 10 (CXCL10), is part of the innate immune response and functions in drawing leukocytes to sites of infection [51]. This increase in plasma CXCL10 concentrations as a consequent of LPS injection has been noted before in sheep [52]. In the current study, IP-10 significantly increased between h 0 and 4, again demonstrating that the ewes successfully mounted an acute-phase response against the LPS endotoxin.

Systemic recognition of LPS endotoxin facilitates the synthesis of acute-phase proteins, such as haptoglobin, by the liver [47], which was also validated in this study. Haptoglobin helps to initiate an anti-inflammatory response after stimulation with LPS through reducing the expression of inflammatory factors in macrophages; Haptoglobin also sequesters iron so it is unavailable for bacterial use, thus preventing oxidative damage [53–55]. Contrarily, the acute inflammation caused by LPS is known to reduce levels of negative acute-phase proteins, such as albumin [49], which was also observed in the present study. Interestingly, the 0.3 mg iSe group had significantly lower albumin concentrations in comparison to the oSe treatments at h 4. In addition to selenoprotein P secreted by the liver and extracellular GPx, albumin is another known transporter of Se [56]. In humans, one study found that a positive correlation between serum inorganic Se concentrations and Se bound to albumin, indicating that albumin may play a key role in transporting this type of Se throughout the body [56]. However, the exact reason why albumin concentrations would significantly decrease in the iSe treatment in comparison to the oSe treatments is unknown, unless there was increased inflammation experienced by animals in the iSe group [12, 57]. Just as with albumin, the total protein and globulin concentrations also significantly decreased after h 0 of the LPS challenge. However, the ewes in the 0.3 mg iSe treatment group had total protein and globulin concentrations that fell below the reference intervals for those parameters denoted by the University of Guelph’s Animal Health Laboratory (65–90 g/L and 26–52 g/L, respectively) unlike the ewes from the other treatment group. Overall, the drop in albumin, total protein, and globulin concentrations between h 0 and 4 was markedly more noticeable in the 0.3 mg iSe group, and this decline was significant when comparing the 0.3 mg iSe to one or more of the other treatments. To the authors’ knowledge, no other research has reported these finding, but they may be reflective of increased levels of oxidative stress induced by the iSe treatment [58]. In comparison to oSe, iSe can more easily be converted into pro-oxidant selenocompounds such as hydrogen selenide (H_2_Se) or selenols, which can be oxidized and generate ROS; in cases of heightened ROS production, iSe toxicity can lead to various consequences such as DNA damage, protein degradation, lipid peroxidation and apoptosis [58]. Additionally, serum cholesterol and glucose levels significantly rose at h 4 post-LPS injection for most treatment groups, corresponding to the findings of another study in poultry showing that an oxidative stress challenge induced a rise in blood cholesterol and glucose [59]. A study in sheep showed a similar elevation in blood glucose levels at h 2 post-LPS injection [52]. This effect may be particularly due to the concurrent rise in cortisol concentrations which induces gluconeogenesis [60]. Interestingly, there were some statistically significant differences at h 4 in serum Na, K, and Cl concentrations when comparing across treatment groups. For all these parameters, the control group had significantly different ion concentrations than one or more of the other Se supplemented treatment groups. However, the mean concentrations were all within the normal ovine physiological range, so it is unclear whether these findings are biologically relevant. The ewe’s Na:K ratio increased significantly from time 0 to time 4 in all treatment groups except the 0.3 mg iSe group, which may be due to the transient increase in Na retention and K excretion induced by the simultaneous increase in cortisol concentrations [60]. During the acute phase response prompted by LPS endotoxin, the rise of cortisol concentrations was in agreement with previous studies [48]. Finally, ewe rectal temperatures also significantly increased in the hours post-LPS injection, as demonstrated in others studies [52, 61].

## Conclusion

Overall, the 0.3 mg oSe supplementation group maintained higher serum Se concentrations in comparison to the 0.3 mg iSe group after 40 d, highlighting the potential benefit of using an oSe rather than iSe supplementation strategy for better long term improvements in serum Se status. However, no benefit was found in supplementing with 0.6 mg oSe over 0.3 mg oSe in terms of serum Se status. Additionally, no major differences in innate or adaptive immunological responses were detected during the supplementation period. Future studies should investigate whether supranutritional Se supplementation over a longer period of time is needed to confer immunological benefits or if simply supplementing the minimal amount to prevent deficiency is sufficient for adequate immune function.

## Supplementary Information

Additional file 1: Supplemental Table S1. Ingredients and nutrients in the ewe's pelleted feed. Supplemental Table S2. A summary of parameters measured in the ovine biochemistry profile.

## Data Availability

The datasets used and/or analysed during the current study are available from the corresponding author on reasonable request.

## Abbreviations

AHL: Animal Health Laboratory
AIC: Akaike Information Criterion
BCS: Body condition score
CIDR: Controlled internal drug release
CV: Coefficient of variation
DHR: Dermal hypersensitivity response
ELISA: Enzyme-linked immunoassay
GD: Gestation day
GPx: Glutathione peroxidase
GSH: Glutathione
GSSG: Glutathione disulfide
ICP-MS: Inductively couples plasma mass spectrometry
IFN: Interferon
Ig: Immunoglobulin
IL: Interleukin
IP-10: Interferon gamma-induced protein 10
iSe: Inorganic Se
LPS: Lipopolysaccharide
LSM: Least squares means
NADPH: Nicotinamide adenine dinucleotide phosphate
NK: Natural killer
oSe: Organic Se
OVA: Ovalbumin
PBS: Phosphate-buffered saline
PPD: Postpartum day
S: Sulfur
Se: Selenium
SeCys: Selenocysteine
SeMet: Selenomethionine
TMB: 3,3′,5,5′-Tetramethylbenzidine
