# Supplementary material for: Effect of different sources of selenium supplementation on immune function in pregnant and lactating ewes
Source: J Anim Sci Biotechnol. 2025 Dec 25;16:180. doi: 10.1186/s40104-025-01311-9 (PMC12739852; doi:10.1186/s40104-025-01311-9)
Supplement: Supplementary file 1 — Additional file 1: Supplemental Table S1. Ingredients and nutrients in the ewe's pelleted feed. Supplemental Table S2. A summary of parameters measured in the ovine biochemistry profile. [file 40104_2025_1311_MOESM1_ESM.docx]

**Supplemental Table S1** Ingredients and nutrients in the ewe’s pelleted feed

| **Ingredients** | **Percentage of as-is diet** |
| --- | --- |
| Soybean meal (47%) | 34 |
| Wheat shorts | 17 |
| High bypass soybean meal | 10 |
| Hi Pro corn gluten (60%) | 10 |
| Limestone calcium carbonate | 7.46 |
| Soy hulls (Ground) | 5 |
| Canola | 5 |
| Fine salt | 3.16 |
| Ammonium chloride | 2.2 |
| Monocalcium phosphate (DICAL) | 1.89 |
| Vitamin E (50 kIU/kg) | 1.81 |
| Tallow (AV blend) | 1 |
| Magnesium oxide | 0.55 |
| PellTech^TM^ | 0.4 |
| Lasalocid | 0.09 |
| Selenium free mineral mix^*^ | 0.44 |
| **Chemical composition, as-is basis** | Unit specified |
| Total digestible nutrients, % | 60.84 |
| Net energy for maintenance, Mcal/kg | 1.52 |
| Net energy for growing, Mcal/kg | 1.04 |
| Net energy for lactation, Mcal/kg | 1.44 |
| Non-structural carbohydrates, % | 19.63 |
| Starch, % | 6.87 |
| Crude protein, % | 34.85 |
| Undegradable intake protein, % | 12.21 |
| Degradable intake protein, % | 19.06 |
| Soluble protein, % | 9.28 |
| Equivalent crude protein/nonprotein nitrogen compounds, % | 3.58 |
| Lysine, % | 1.59 |
| Methionine, % | 0.48 |
| Crude fat, % | 3.21 |
| Crude fibre, % | 5.43 |
| Acid detergent fibre, % | 7.08 |
| Neutral detergent fibre, % | 12.71 |
| Dry matter, % | 90.11 |
| Calcium total, % | 3.41 |
| Phosphorous total, % | 0.96 |
| Ash, % | 16.68 |
| Sodium, % | 1.27 |
| Chloride, % | 3.34 |
| Potassium, % | 1.24 |
| Magnesium, % | 0.58 |
| Sulphur, % | 0.39 |
| Iron, Mcal/kg | 729.65 |
| Manganese, Mcal/kg | 249.12 |
| Zinc, Mcal/kg | 556.70 |
| Copper, Mcal/kg | 4.86 |
| Iodine, Mcal/kg | 5.28 |
| Cobalt, Mcal/kg | 1.41 |
| Fluorine, Mcal/kg | 34.02 |
| Vitamin A, KIU/kg | 44 |
| Vitamin D_3_, KIU/kg | 11 |
| Vitamin E, IU/kg | 1,103 |
| Dietary cation-anion difference, Meq/kg | -311.83 |
| Lasalocid sodium, Mg/kg | 180 |

*Selenium free mineral mix is 98% DM and contains 100,000 mg/kg iron, 50,000 mg/kg manganese, 120,000 mg/kg zinc, 1,200 mg/kg iodine, 300 mg/kg cobalt, 10,000 kIU/kg Vitamin A, 10,000 kIU/kg vitamin D_3_, 45,000 IU/kg Vitamin E

**Supplemental Table S2** A summary of parameters measured in the ovine biochemistry profile

| **Parameter** | **Reference interval** | **Indicator** |
| --- | --- | --- |
| Calcium | 2.43–2.91 mmol/L | Dietary imbalance, disease |
| Phosphorous | 1.1–2.54 mmol/L |  |
| Magnesium | 0.89–1.28 mmol/L |  |
| Sodium | 143–153 mmol/L | Electrolyte balance |
| Potassium | 4–5.5 mmol/L |  |
| Chloride | 102–113 mmol/L |  |
| Sodium:Potassium ratio | N/A |  |
| Calculated osmolality | N/A | Fluid status |
| Haptoglobin | 0–0.8 g/L | Inflammation, disease |
| Urea | 3.4–12.0 mmol/L | Kidney function, protein metabolism |
| Creatinine | 28–100 mmol/L |  |
| Cholesterol | 1.13–2.57 mmol/L | Lipid metabolism, metabolic health |
| Non-esterified fatty acids | 0–0.3 mmol/L |  |
| Glutamate dehydrogenase | 0–25 U/L | Liver function, inflammation |
| Total bilirubin | 0–2 mmol/L |  |
| Alkaline phosphatase | 0–247 U/L |  |
| Gamma-glutamyl transferase | 17–77 U/L |  |
| Albumin | 29–47 g/L | Liver function, nutritional status |
| Total protein | 65–90 g/L |  |
| Globulin | 26–52 g/L |  |
| Albumin:Globulin ratio | N/A |  |
| Aspartate aminotransferase | 64–158 U/L | Liver pathology, inflammation |
| Anion gap | N/A | Metabolic acidosis |
| Glucose | 2.6–4.4 mmol/L | Metabolic health |
| Beta-hydroxybutyrate | 185–605 mmol/L |  |
| Creatine kinase | 23–313 U/L | Muscle function, cardiac function, neurological function |
| Carbon dioxide | N/A | Rumen function |

Reference intervals were defined by the Animal Health Laboratory (University of Guelph, Guelph, ON)
